# Exploring Applications of Transcranial Magnetic Stimulation in Child and Adolescent Psychiatry: A Narrative Review

**DOI:** 10.3390/jcm14186513

**Published:** 2025-09-16

**Authors:** Omar El-Shahawy, Mona Salehi, Mahdieh Saeidi, Sanobar Jaka, Julia Lopez, Pegah Yakhchalian, Mohsen Abbasi-Kangevari, Sasidhar Gunturu

**Affiliations:** 1 Department of Population Health, Section for Tobacco, Alcohol and Drug Use, New York University Grossman School of Medicine, New York, NY 10016, USA; jsanobar@gmail.com (S.J.); mohsen.abbasikangevari@nyulangone.org (M.A.-K.); 2 School of Global Public Health, New York University, New York, NY 10003, USA; 3 Department of Psychiatry, Bronx Care Health System, New York, NY 10456, USA; saleh109@umn.edu (M.S.); msaeidi@bronxcare.org (M.S.); jlopez9@bronxcare.org (J.L.); sguntur1@bronxcare.org (S.G.); 4 Department of Psychiatry, University of Minnesota School of Medicine, Minneapolis, MN 55455, USA; 5 Department of Psychiatry, Johns Hopkins University School of Medicine, Baltimore, MD 21287, USA; 6 Department of Psychiatry and Behavioral Sciences, Nassau University Medical Center, East Meadow, NY 11554, USA; 7 Department of Psychiatry and Behavioral Sciences, Kaweah Delta Health Care District, Visalia, CA 93277, USA; pegyakhc@kaweahhealth.org; 8 Department of Psychiatry, Icahn School of Medicine at Mount Sinai, New York, NY 10029, USA

**Keywords:** transcranial magnetic stimulation (TMS), child psychiatric disorders, adolescent psychiatric disorders, neuromodulation

## Abstract

**Background**: Transcranial magnetic stimulation (TMS) is an emerging noninvasive treatment modality for various psychiatric disorders, but its applications in child and adolescent populations remain underexplored. This review aims to synthesize current evidence on the therapeutic potential of TMS in treating psychiatric conditions within this demographic. **Methods**: A comprehensive literature search was performed using PubMed and Google Scholar databases to identify studies published up to March 2025 that reported on the use of transcranial TMS in child and adolescent psychiatric disorders. **Results**: We found 32 published studies that included at least one type of TMS. TMS demonstrates potential as a safe and effective intervention for conditions such as depression, autism spectrum disorder, attention-deficit/hyperactivity disorder, obsessive–compulsive disorder, Tourette Syndrome, and childhood schizophrenia. However, the therapeutic outcomes vary significantly across conditions and protocols. **Conclusions**: TMS offers a promising, well-tolerated option for addressing psychiatric disorders in children and adolescents, but its application requires careful ethical and clinical consideration. To fully realize its potential, future research should focus on refining protocols, standardizing methodologies, and ensuring safety while expanding its use across diverse psychiatric conditions in younger populations.

## 1. Introduction

The worldwide-pooled prevalence of any mental disorder among children and adolescents aged 5 to 24 years is estimated at 11.63%, according to the 2019 Global Burden of Disease study. This includes specific prevalence rates for various diagnostic groups, such as anxiety disorders (3.35%), depressive disorders (1.87%), attention-deficit/hyperactivity disorder (2.22%), and conduct disorder (2.18%). There is a profound burden that these mental disorders place on children, adolescents, families, and society. These disorders can negatively impact various aspects of individuals’ lives, such as academic performance, social relationships, and overall well-being, often leading to emotional and financial strain on families due to treatment costs [1]. There have been substantial advances in psychopharmacological treatments over the past decades. Despite advances in psychopharmacology, challenges remain as no single medication is universally effective, and safety concerns such as side effects and long-term risks limit their utility [2]. Biomarker research has also advanced with many modalities, but few of these tools can dynamically probe the physiology of neurological pathways and synaptic functions [3].

In addressing these challenges, the utilization of innovative modalities like Transcranial Magnetic Stimulation (TMS) is becoming increasingly important. TMS is a non-invasive method of stimulating brain tissue through the production of high or low-intensity magnetic fields, which modulate cortical excitability [4]. Barker and colleagues introduced contemporary TMS devices in Sheffield, England, in 1985, and demonstrated the ability to probe and stimulate the human motor cortex [5].

TMS was introduced as a potential treatment for treatment-resistant major depressive disorder (MDD), with early clinical trials exploring its antidepressant effects [6]. TMS generates an electric field in the brain that modulates neuronal activity by influencing both excitatory and inhibitory firing patterns. It primarily targets cortical gyri and underlying white matter, with effects spreading across local and distant neural networks, including the peripheral sensory and arousal systems. Over the past decades, TMS has been studied in a range of psychiatric disorders, particularly in adults, including major depressive disorder (MDD), autism spectrum disorder (ASD), attention-deficit/hyperactivity disorder (ADHD), Tourette syndrome (TS), schizophrenia, and suicidal ideation [7,8,9,10,11,12].

TMS offers precise neuromodulation without the systemic effects associated with pharmacological interventions by directly targeting specific brain regions implicated in various psychiatric and neurological disorders [13]. Despite growing interest and several published randomized controlled trials demonstrating the efficacy and safety of TMS in children, the underlying mechanisms and optimal protocols remain investigational. The existing literature offers a diverse body of studies employing a range of methodologies, sample sizes, and outcome measures, reflecting the field’s exploratory nature and highlighting opportunities for further standardization to inform clear clinical guidelines.

This review evaluates the current evidence on TMS for child and adolescent psychiatric disorders. Although preliminary findings are encouraging, evidence is still preliminary because many trials have used small samples and heterogeneous methods. We aim to synthesize existing data, identify knowledge gaps, and guide future research to optimize TMS protocols and enhance its clinical utility for younger populations.

## 2. Materials and Methods

A comprehensive literature search was conducted using PubMed and Google Scholar through the end of March 2025 to identify studies reporting the therapeutic use of TMS in child and adolescent psychiatric disorders. As this is a narrative review, two authors independently conducted the search. Only articles published in English were included.

Search terms included: “Transcranial Magnetic Stimulation,” “TMS,” “pediatric psychiatric disorders,” “child psychiatry,” “adolescent psychiatry,” “neuromodulation,” along with disorder-specific terms such as “ADHD,” “autism spectrum disorder (ASD),” “Tourette syndrome,” “obsessive–compulsive disorder (OCD),” “childhood schizophrenia,” “post-traumatic stress disorder (PTSD),” “suicidal ideation,” and “mood disorder”. Reference lists of included articles were manually reviewed to identify additional relevant studies. Gray literature was excluded.

Inclusion criteria were: (1) peer-reviewed original research articles, reviews, or case series; (2) studies involving participants aged 18 years or younger; (3) studies reporting on the therapeutic application of TMS for any psychiatric disorder; and (4) studies providing outcome data on efficacy, safety, or tolerability. Exclusion criteria included: (1) studies not specific to pediatric or adolescent populations; (2) articles without available full text; (3) conference abstracts, editorials, commentaries, or opinion pieces; and (4) non-English publications. We limited the lower bound of the search to 1 January 2001 because the first peer-reviewed clinical case series on pediatric TMS [14] was published that year, marking the onset of systematic investigation in this population. Earlier work consisted only of technical descriptions in adults and animal models with no pediatric clinical data.

## 3. Results

This section is divided into findings regarding the TMS types (Table 1) and their clinical relevance, as well as the list of studies that reported using any type of TMS (Table 2).

### 3.1. Overview of Types of TMS

There is a variety of TMS techniques, each with its own characteristics and applications, including single-pulse TMS (sTMS), paired-pulse TMS, paired associative stimulation (PAS), repetitive TMS (rTMS), theta-burst stimulation (TBS), deep TMS, sequential bilateral approaches, and accelerated TMS. Although these TMS techniques were initially developed and primarily studied in adult populations, this review explores their application in children and adolescents. The goal is to assess their safety and efficacy in treating psychiatric disorders within these younger age groups (Table 1).

#### 3.1.1. Single-Pulse Transcranial Magnetic Stimulation (sTMS)

sTMS involves delivering a single magnetic pulse to a specific brain region and is most commonly used for neurophysiological mapping and measuring cortical excitability. Clinically, it plays a critical role in determining the resting motor threshold (RMT)—the minimum stimulation intensity needed to elicit a motor-evoked potential (MEP) of at least 50 µV in a target muscle in 50% of trials [15]. This measurement is foundational in establishing individualized treatment parameters, particularly in therapeutic rTMS and TBS protocols [15]. The MEP is typically elicited by stimulating the primary motor cortex, which activates the corticospinal tract and induces muscle contraction. Standard sTMS procedures involve single pulses delivered at intervals of 4–8 s [16]. Clinical Relevance: Accurate determination of RMT is essential for safe and effective TMS dosing in both research and clinical applications. In pediatric populations, adjustments to stimulation parameters such as dosage, intensity, electrode size, polarity, and coil placement are essential to ensure safety and efficacy, given the unique anatomical and physiological characteristics of the developing brain [17].

**Table 1:** Comparative Overview of Various Types of Transcranial Magnetic Stimulation (TMS).

TMS Modality	Stimulation Frequency	Intensity (% of Motor Threshold)	Typical Session Duration	Number of Sessions	Total Treatment Duration	Primary Clinical Applications
Repetitive TMS (rTMS)-a [18]	High-Frequency rTMS (Left DLPFC): 10 Hz	80–120% RMT	~20–37.5 min	10–30 sessions	2–6 weeks	Major Depressive Disorder (treatment-resistant), Anxiety, ASD (modest evidence), ADHD
Repetitive TMS (rTMS)-b [18]	Low-Frequency rTMS (Right DLPFC/SMA/LTPC): 1 Hz	90–110% RMT	~15–30 min	10–30 sessions	2–6 weeks	OCD, Tourette Syndrome, Schizophrenia (auditory hallucinations), Depression with irritability
Theta Burst Stimulation (TBS) [18]	Bursts at 50 Hz, repeated at 5 Hz	~80% of Active Motor Threshold	iTBS: ~3 min cTBS: 20–40 s	Typically 8–30 sessions	4 days to 6 weeks	Depression, ASD, Tourette Syndrome (limited studies; ongoing trials)
Deep TMS (H1 Coil) [19]	18 Hz	Up to 120% of MT	55 trains × 2 s, 20-s intertrain interval (1980 pulses)	~20–30 sessions planned	~4–6 weeks	Treatment-Resistant Depression (adolescent trial phase)
Low-Intensity TMS (LI-TMS) [20]	10 Hz	Subthreshold	10 min/session	Standard: 1×/day for 10 days Accelerated: 3×/day for 5 days	1–2 weeks	Preclinical model of adolescent depression; informs future clinical use
Magnetic Seizure Therapy (MST) [21]	100 Hz	100% (train duration titrated)	2–10 s train duration (seizure induced)	Acute: 18 sessions Continuation: 9 sessions	Acute: 6 weeks Continuation: 6 months	Refractory Bipolar Depression (Adolescent case study)
Single pulse TMS (sTMS) [22]	1 pulse per stimulus (not repeated)	Variable (usually suprathreshold, e.g., 120%)	Milliseconds (used for motor evoked potentials or silent periods)	1	N/A (Research use only)	Neurophysiological assessment: cortical excitability, inhibition (e.g., CSP, MEP), biomarker research
Paired Pulse TMS (ppTMS) [22]	Two pulses: Conditioning + Test (1–200 ms ISI)	Conditioning: subthreshold; Test: suprathreshold (e.g., 80–120%)	~30–60 min (research session)	1 (research setting)	N/A (used as assessment)	Assessment of cortical excitability and inhibition (e.g., SICI, ICF, LICI); research in ADHD, ASD, depression

Abbreviations: RMT: resting motor threshold; MT: motor threshold; iTBS: intermittent theta-burst stimulation; cTBS: continuous theta-burst stimulation; OCD: obsessive–compulsive disorder; ASD: autism spectrum disorder; ADHD: attention-deficit/hyperactivity disorder.

**Table 2:** Published Studies of TMS in Psychiatric Disorders Among Children and Adolescent Populations.

Title	Authors	Type of Study	Sample Size	Psychiatric Disorder	Stimulation Target/Site	Protocols	Key Findings (Effect Size ± *p*)	Scales (*p*-Value)	Evidence Level
Repetitive transcranial magnetic stimulation (rTMS) in the treatment of obsessive–compulsive disorder (OCD) and Tourette syndrome (TS)	Mantovani et al., 2006 [23]	Open-label pilot study	10	OCD and TS	Supplementary Motor Area	Four daily trains at 1 Hz, 100% RMT, 5 min duration, 2 min inter-train interval (1200 stimuli/day)	OCD group (n = 7): Significant reduction in OCD symptoms (YBOCS ↓ 36.4 → 26, *p* = 0.007). TS group (n = 5): Significant reduction in tic severity (YGTSS ↓ 71.2 → 23.4, *p* = 0.024). Overall sample (N = 10): Marked improvement in CGI (*p* < 0.001, sustained at 3 months), anxiety (HARS ↓ 24.1 → 12, *p* < 0.001), and depression (HDRS ↓ 20.7 → 10.8, *p* = 0.001; BDI ↓ 10 → 6.4, *p* = 0.022). Right motor threshold increased significantly.	YBOCSYGTSS, HDRS-24, HARS-1, BDI SASS	Level IV (Open-label pilot study with no control group)
An 11-year-old boy with drug-resistant schizophrenia treated with temporo-parietal rTMS	Jardri et al., 2007 [24]	Case report	1	Schizophrenia	Left temporo-parietal cortex	Low-frequency (1 Hz) fMRI-guided rTMS over the left temporo-parietal cortex, 10 sessions, repeated every 5 weeks	47% reduction in auditory verbal hallucinations (*p*-value not reported) 40% improvement in adaptive functioning (*p*-value not reported)	Auditory Hallucinations Rating Scale, Children’s Global Assessment Scale	Level V (5): Case report or expert opinion
Repetitive transcranial magnetic stimulation for the treatment of obsessive–compulsive disorder: a double-blind controlled investigation	Sachdev et al., 2007 [25]	RCT, double-blind placebo-controlled study	18	OCD	Not specified	30 trains of 5 s each, at 10 Hz and 110% motor threshold, with 25 s inter-train intervals (1500 stimuli per session)	No significant difference between rTMS and sham after 2 weeks. Within-subject improvement in OCD symptoms after 4 weeks, but not significant after adjusting for depression. rTMS improved depressive symptoms (MADRS). Well tolerated, only mild transient side effects.	YBOCS obsession, YBOCS compulsion Maudsley Obsessive-Compulsive Inventory scores	Level II (Evidence from at least one well-designed randomized controlled trial)
Effectiveness of the repetitive Transcranial Magnetic Stimulation (rTMS) of 1 Hz for Attention-Deficit Hyperactivity Disorder (ADHD)	Niederhofer et al., 2008 [26]	Case report	1	ADHD	Not specified	Low frequency (1 Hz, 1200 stimulations per day for five days)	Marked improvement in ADHD symptoms (hyperactivity, impulsivity, attention). Effects sustained for at least 4 weeks post-treatment. Placebo condition showed no improvement. No effect size or *p*-values reported.	Conner’s rating scale for adult	Level V (5): Case report or expert opinion
The effect of repetitive transcranial magnetic stimulation on symptoms in obsessive–compulsive disorder	Prasko et al., 2006 [27]	RCT, double-blind, sham-controlled study	37	OCD	Left DLPFC	10 sessions of rTMS, 1 Hz at 110% of motor threshold	No significant difference between rTMS and sham groups on OCD symptoms (Y-BOCS). No group differences on other scales (CGI, HAMA, BAI). rTMS was safe and well tolerated.	CGI, HAMA, Y-BOCS BAI	Level II (randomized, double-blind, sham-controlled trial)
Effects of low frequency repetitive transcranial magnetic stimulation (rTMS) on gamma frequency oscillations and event-related potentials during processing of illusory figures in autism	Sokhadze et al., 2009 [28]	Open-label	13	ASD	DLPFC	Two times per week for 3 weeks (a total of six 0.5 Hz rTMS treatments) over the left DLPFC. Stimulation at 0.5 Hz and 90% MT, with a total of 150 pulses/day	Significant improvement in ASD-related symptoms over time. No significant difference between active rTMS and sham groups. Well tolerated; no adverse events reported.	Caregiver report and clinician ratings of improvement, Aberrant Behavior Checklist (ABC), Social Responsiveness Scale (SRS), CGI	Level II (Randomized, double-blind, sham-controlled trial)
Low-Frequency Repetitive Transcranial Magnetic Stimulation (rTMS) Modulates Evoked-Gamma Frequency Oscillations in Autism Spectrum Disorder (ASD)	Baruth et al., 2010 [29]	RCT	25	ASD	Left DLPFC	rTMS, 1 Hz, 90% RMT, 10 min	Reduced gamma-band cortical activity. Improvement in behavioral symptoms (ABC, RBS-R). Suggests modulation of cortical excitability and inhibitory control.	Repetitive behavior scale–revised (RBS) (*p* = 0.02). Social responsiveness scale (SRS) (N.S). Aberrant behavior checklist (ABC) (*p* = 0.002).	Level IV (Open-label, uncontrolled study)
Noninvasive brain stimulation with high-frequency and low-intensity repetitive transcranial magnetic stimulation treatment for posttraumatic stress disorder	Boggio et al., 2010 [30]	Double-blind, placebo-controlled phase II trial	30	PTSD	DLPFC	10 daily sessions over 2 weeks of active 20 Hz rTMS of the right DLPFC, active 20 Hz rTMS of the left DLPFC, or sham rTMS	Both right and left DLPFC rTMS groups improved significantly in PTSD symptoms. Right DLPFC stimulation produced greater benefit on core PTSD symptoms and anxiety. Left DLPFC stimulation produced greater improvement in mood. Effects persisted at 3-month follow-up. Safe and well tolerated.	PTSD Checklist; Treatment Outcome PTSD Scale; Hamilton Anxiety Rating Scale; Hamilton Depression Rating Scale	Level II (Randomized, double-blind, sham-controlled trial)
1 Hz low frequency repetitive transcranial magnetic stimulation in children with Tourette syndrome	Kwon et al., 2011 [31]	Open-label trial	10	TS [ADHD, MDD, OCD]	SMA	rTMS, supplementary motor area (SMA), 10 sessions, 1 Hz rTMS, 100% of motor threshold, 1200 stimuli/day	Significant reduction in tic severity (YGTSS ↓ from 20.6 to 13.5, *p* = 0.012). Significant improvement in CGI-TS scores (*p* = 0.002). No significant effects on ADHD, depression, or anxiety measures.	Yale Tic Rating Scale; CGI-TS	Level IV (case series without control group)
Repetitive Transcranial Magnetic Stimulation (rTMS) Modulates Event-Related Potential (ERP) Indices of Attention in Autism	Casanova et al. monitoring, 2012 [32]	RCT	45	ASD	SMA	1 Hz and 90% MT, 150 pulses/day (15 × 10 s trains with 20 at 30 s intervals). 12 sessions: 6 left and 6 right DLPFC	Significant reduction in tic severity (YGTSS, *p* = 0.012). Improvement in CGI-TS scores (*p* = 0.002). No significant changes in ADHD, anxiety, or depression scores.	Aberrant Behavior Checklist (ABC; Social Responsiveness Scale (SRS); Repetitive Behavior Scale (RBS)	Level IV (Open-label pilot cohort study)
A randomized, double-blind trial of repetitive transcranial magnetic stimulation in obsessive–compulsive disorder with three-month follow-up	Gomes et al., 2012 [33]	RCT	22	OCD [MDD]	pre-SMA, bilaterally	1 Hz, 20 min trains (1200 pulses/day) at 100% of resting MT, once per day, 5 days/week, for 2 weeks	At 2 weeks: 42% responders in active vs. 12% in sham (*p* < 0.001). At 14 weeks: 35% responders in active vs. 6% in sham (*p* < 0.001). Significant improvements in CGI-S and anxiety (HAM-A). No significant effect on depression.	Y-BOCS; HAMD-24; BDI–IIc, HARS-14, CGI–S	Level II (Randomized, double-blind, sham-controlled trial)
Transcranial magnetic stimulation (TMS) in the treatment of attention-deficit/hyperactivity disorder in adolescents and young adults: a pilot study	Weaver et al., 2012 [34]	RCT, sham-controlled, crossover	9	ADHD [Anxiety disorders]	Right DLPFC	rTMS at 10 Hz, 100% motor threshold, 2000 pulses/session, 10 sessions/2 weeks	CGI-I: Significant overall improvement across phases (*p* < 0.005). ADHD-IV: 9-point reduction overall (*p* < 0.05), but no difference between active and sham conditions. Neurocognitive & EEG outcomes: no group differences. Well tolerated (mild headache/scalp discomfort in 3 participants).	CGI-I scale, ADHD-IV scale	Level II (Randomized, sham-controlled crossover pilot study)
Transcranial magnetic stimulation at 1 Hertz improves clinical symptoms in children with Tourette syndrome for at least 6 months	Le et al., 2013 [35]	Clinical trial	25	TS [ADHD, anxiety, and depression]	Right prefrontal cortex (approx. right DLPFC, 5 cm anterior to motor hotspot)	1 Hz rTMS, 20 daily sessions, 5 days/week × 4 weeks, 110% RMT, 1200 stimuli/day	Significant improvement in tic severity (CGI-I, *p* < 0.005). ADHD symptoms reduced (ADHD-IV, *p* < 0.05), though no significant sham difference. Mean treatment effect size = 0.48 (not statistically significant).	YGTSS; CGI; SNAP-IV; Kovacs’ CDI; SCAS	Level II
Effects of weekly low-frequency rTMS on autonomic measures in children with autism spectrum disorder	Casanova et al., 2014 [36]	Clinical trial	18	ASD	DLPFC	rTMS, 5 Hz, 90% resting motor threshold, 10 min duration, 18 sessions, 160 pulses delivered, trains with 8.10 s on, 20 s ISI, Right and Left DLPFC	Significant reduction in repetitive and ritualistic behaviors (RBS-R). Improvements in irritability, lethargy, and hyperactivity (ABC subscales). Overall behavioral benefits with good tolerability.	Aberrant Behavior Checklist, Restricted Behavior Pattern	Level 3 (non-randomized, open-label, proof-of-concept study)
Low frequency repetitive transcranial magnetic stimulation in children with attention deficit/hyperactivity disorder. Preliminary results	Gómez et al., 2014 [37]	Open-label, preliminary study without a control group	13	ADHD	Left DLPFC	1 Hz rTMS over the Left DLPFC, 5 consecutive days; 1500 stimuli per session, 90% of resting motor threshold	Significant improvement in ADHD symptoms (parent and teacher checklists). Strongest effects in inattention at school and hyperactivity/impulsivity at home. Well tolerated (headache, local discomfort, mild neck pain; no seizures or cognitive changes).	Checklist (SCL) for ADHD from DSM-IV	Level 4 (Open-label, uncontrolled pilot study)
Functional MRI-navigated repetitive transcranial magnetic stimulation over supplementary motor area in chronic tic disorders	Wu et al., 2014 [38]	RCT, double-blind, parallel, Sham-controlled trial	12	TS [ADHD, OCD]	SMA	cTBS, Bilateral SMA/Sham, 2 sessions, fMRI-navigated, (3-pulse 30 Hz bursts, repeated 5 times/s, 90% RMT, 600 pulses/train, 4 trains/day)	Active cTBS reduced SMA and motor cortex activation on fMRI compared with sham. No significant clinical difference in tic severity (YGTSS) between active and sham groups. Suggests neurophysiological modulation without clear clinical benefit.	YGTSS; PUTS; CYBOCS	Level II Randomized, double-blind, sham-controlled trial
rTMS neuromodulation improves electrocortical functional measures of information processing and behavioral responses in autism	Sokhadze et al., 2014 [39]		27	ASD	DLPFC	1 Hz frequency, 90% MT, 180 pulses/day, 9 trains of 20 pulses each, 20–30 s inter-train interval	EEG: Reduced P50/P300 amplitudes, increased N100/N200 amplitudes (improved cortical processing). Behavior: Significant reductions in irritability, hyperactivity, stereotypy (ABC subscales). Suggests improvements in both neurophysiological and behavioral outcomes.	Aberrant Behavior Checklist (ABC), Repetitive Behavior Scale (RBS-R)	Level III, Open-label study with no sham control; small sample size
A simple, repeated rTMS protocol effectively removes auditory verbal hallucinations in a single patient study	Blanco-Lopez et al., 2016 [40]	Case report	1	Schizophrenia	Left Temporoparietal Cortex	Two blocks of 10 daily sessions over two weeks each; low-frequency (1 Hz) rTMS over the left temporoparietal cortex, 1200 stimuli/session at 90% RMT	Complete and sustained resolution of auditory verbal hallucinations after second treatment block. Depression scores improved (PHQ-9: 23 → 7). Grief and psychosis ratings also improved (ICG: 5 → 1).	PHQ-9, ICG, PYSRATS	Level IV, case report, no control group
Clinical effects of repetitive transcranial magnetic stimulation combined with atomoxetine in the treatment of attention-deficit hyperactivity disorder	Cao et al., 2018 [41]	RCT	64	ADHD	Right dorsolateral prefrontal cortex	10 Hz, 4 s stimulation, 26 s interval, 100% rMT, 2000 pulses/session, 25 min/session, 5 sessions/week for 6 weeks (30 sessions total)	Significant symptom improvements across all groups (rTMS, atomoxetine, and rTMS + ATX). Combination group (rTMS + ATX) showed greatest improvements in attention, hyperactivity/impulsivity, and oppositional behaviors. Executive function (digit span, arithmetic, coding, CPT, IGT) improved most in the combination group. Well tolerated.	SNAP-IV continuous performance test, WISC (arithmetic, and coding	Level II
High-frequency repetitive TMS for suicidal ideation in adolescents with depression	Croarkin et al., 2018 [12]	Open-label	19	Suicidal ideation [MDD]	Left dorsolateral prefrontal cortex	rTMS, Left DLPFC, 30 sessions, 10 Hz, 120% motor threshold, 4 s trains, 26 s intertrain, 3000 pulses/session	Significant reduction in suicidal ideation (C-SSRS, CDRS-R Item 13). However, effects lost significance after adjusting for depression severity. Treatment was well tolerated.	C-SSRS CDRS-R Item 13	Level: III (Exploratory open-label study)
Exploratory Study of rTMS Neuromodulation Effects on Electrocortical Functional Measures of Performance in an Oddball Test and Behavioral Symptoms in Autism	Sokhadze et al., 2018 [42]		124	ASD	Dorsolateral Prefrontal Cortex	1 Hz frequency, 90% MT, 180 pulses/session (9 trains of 20 pulses each with 20–30 s intervals)	Significant improvements in error monitoring (↓ commission errors, improved post-error slowing). ERP changes: more negative ERN amplitude, shorter ERN latency, changes in P3a/P3b. Behavioral benefits: reductions in ritualistic behaviors, stereotypy, irritability, hyperactivity, and lethargy (ABC, RBS-R).	Aberrant Behavior Checklist (ABC) (RBS-R):	Level II
Does rTMS reduce depressive symptoms in young people who have not responded to antidepressants?	Rosenich et al., 2019 [43]	Retrospective study	15	Treatment-resistant MDD [Various psychiatric comorbidities]	Right dorsolateral prefrontal cortex (DLPFC) in unilateral protocols; both right and left DLPFC in bilateral protocols	Bilateral or unilateral rTMS, 6-week protocol	Significant improvements in depression scores (HAM-D, MADRS, Zung). Response rate: 40%; remission rate: 13%; partial response in 87%. Well tolerated in a real-world clinical setting.	HAM-D, MADRS	Level IV (Retrospective open-label naturalistic study; no control group or randomization)
Add-on rTMS for the acute treatment of depressive symptoms is probably more effective in adolescents than in adults: Evidence from real-world clinical practice	Zhang et al., 2019 [44]	Naturalistic, observational study	117 (42 adolescents, 75 adults)	Depression and anxiety disorders [Bipolar II, dysthymia, GAD, eating disorder, OCD]	Left dorsolateral prefrontal cortex	rTMS to left DLPFC, 10 Hz, 120% motor threshold, 10–20 sessions	All age groups improved significantly in depressive symptoms. Adolescents showed greater reduction in depression scores and higher response/remission rates compared to adults. Suggests enhanced efficacy of rTMS in adolescent patients.	HAMD, HAMA	Level III (Naturalistic observational study; non-randomized, non-controlled)
Treatment of Executive Function Deficits in autism spectrum disorder with repetitive transcranial magnetic stimulation: A double-blind, sham-controlled, pilot trial	Ameis et al., 2020 [9]	RCT, blinded, parallel, sham-controlled design	40	ASD [ADHD]	Left dorsolateral prefrontal cortex	4-week course of 20 Hz rTMS targeting DLPFC (90%RMT) vs. sham	No significant overall improvement in executive function vs. sham. Exploratory analysis: patients with lower baseline adaptive functioning showed greater benefit from active rTMS. Safe and feasible in ASD youth and young adults.	BRIEF-SR or BRIEF-Adult; CANTAB SWM total errors	Level II (Randomized controlled trial)
Left prefrontal transcranial magnetic stimulation for treatment-resistant depression in adolescents: a double-blind, randomized, sham-controlled trial	Croarkin et al., 2021 [45]	RCT, sham-controlled trial	112	MDD	Left dorsolateral prefrontal cortex	10-Hz, left prefrontal, 30 treatment sessions over 6 weeks, no more than 2 days between sessions	Clinically meaningful reduction in depression (HAM-D-24), but no significant difference between active and sham groups. Response rates: 41.7% (active) vs. 36.4% (sham). Remission rates: ~29% in both groups. Meta-analysis including this and adult trials showed small, non-significant effect.	HAM-D-24	Level I (Randomized, double-blind, sham-controlled trial; multicenter)
Bilateral transcranial magnetic stimulation of the supplementary motor area in children with Tourette syndrome	Kahl et al., 2021 [46]	Open-label trial	10	TS [ADHD, anxiety, social phobia, OCD, epilepsy]	Bilateral Supplementary Motor Area	rTMS, Bilateral SMA (each hemisphere separately), 15 sessions, 1 Hz rTMS, 100%RMT, 900 pulses per hemisphere	Significant reduction in tic severity (YGTSS ↓ from 64.4 → 26.4, *p* = 0.005). Improvements in motor and phonic tic subscores, impairment, anxiety (MASC-2), and depression (CDRS-R). Physiological measures: longer cortical silent period, increased intracortical facilitation. Well tolerated; some MRS data inconclusive.	YGTSS; MASC 2; CDRS-R	Level IV (Open-label, uncontrolled pilot trial)
A lack of efficacy of continuous theta burst stimulation over the left dorsolateral prefrontal cortex in autism: A double blind randomized sham-controlled trial	Ni et al., 2021 [47]	RCT, parallel, double-blind and sham-controlled trial	60	ASD [ADHD, OCD, Learning disorders]	Left dorsolateral prefrontal cortex	cTBS: 3 TMS pulses at 50 Hz at 200 ms intervals for 40 s (600 pulses), 90% AMT	Significant improvements in social communication and interaction (ADOS, SRS). No significant effect on restricted/repetitive behaviors. EEG: increased gamma-band oscillations post-treatment. Well tolerated, no major adverse events.	SRS, RBS-R, Emotion Dysregulation Inventory (EDI)	Level 1b: Randomized controlled trial (RCT), double-blind, sham-controlled
Abnormal individualized functional connectivity: A potential stimulation target for pediatric Tourette syndrome	Wang et al., 2024 [48]	Case–Control Observational study	116	Tourette syndrome	Left Supplementary Motor Area	MRI data were acquired using a 3-T scanner	Children with TS showed significantly lower functional connectivity (FC) between left GPi and left SMA (*p* = 0.011, Bonferroni corrected). Decreased FC was negatively correlated with tic severity (YGTSS, *p* = 0.01; Bonferroni significant). Suggests individualized FC peak in SMA as a potential rTMS target for TS.	Yale Global Tics Severity Scale (YGTSS)	Level III—Prospective observational study
A Multisite, 6-Month, Open-Label Study of Maintenance Transcranial Magnetic Stimulation for Adolescents with Treatment-Resistant Depression	Garzon et al., 2025 [49]	Open-label study	41	Treatment-resistant depression	Left dorsolateral prefrontal cortex	22 TMS session	Maintenance TMS was feasible, safe, and clinically effective. 43% required retreatment; average = 22.5 sessions. Higher baseline depression severity (HAMD-24) predicted greater relapse risk (HR = 1.11, *p* ≤ 0.001). Treatment resistance level (ATR) did not predict relapse.	HAMD-24 CDRS-R: QIDS-SR CGI-S	Level III—Multisite open-label longitudinal follow-up study (non-randomized, uncontrolled)
Function-Specific Localization in the Supplementary Motor Area: A Potential Effective Target for Tourette Syndrome	Wang et al., 2025 [50]	Randomized longitudinal study	54	Tourette syndrome	- Function-specific SMA (left SMA or right SMA) determined by GPi-based peak functional connectivity (FC) - Compared with conventional scalp-localized SMA	cTBS:600 pulses/train (3 pulses/burst at 5 Hz); 3 trains/day for 5 days	Left SMA fMRI-guided targeting: ≥30% YGTSS reduction in 4/19 patients after 1 week. Significant GPi–SMA FC changes correlated with tic reduction (r = 0.638, *p* = 0.026). YGTSS improved significantly (baseline vs. 1 week, *p* < 0.0001; baseline vs. 2 weeks, *p* = 0.005). Demonstrates added value of function-specific SMA targeting vs. conventional scalp-based localization.	YGTSS	Randomized, parallel-group, controlled trial with functional MRI-guided targeting (preliminary evidence; Level II).
Probing the Neurodynamic Mechanisms of Cognitive Flexibility in Depressed Individuals with Autism Spectrum Disorder	Elmaghraby et al., 2025 [51]	Open label Study	9	Autism Spectrum Disorder [TRD]	Dorsolateral prefrontal cortex	30 accelerated theta burst stimulation of thedorsolateral prefrontal cortex, either unilaterally or bilaterally.	rTMS reduced prefrontal excitability and increased right temporoparietal excitability. Significant improvements in fluid cognition (t = 3.79, *p* = 0.005) and depressive symptoms (F(3,21) = 28.49, *p* < 0.001). Cognitive improvement at week 4 correlated with later depression improvement at week 12 (r = 0.71, *p* = 0.05). Suggests early cognitive gains may predict mood response.	NIH Toolbox Fluid Cognition Composite score and the Dimensional Change Card Sort subtest	Open-label pilot study (Level IV)

ABC: Aberrant Behavior Checklist; ADHD_RS: Attention Deficit Hyperactivity Disorder Rating Scale; ADHD-IV: Attention-Deficit/Hyperactivity Disorder Rating Scale; ATR: Antidepressant Treatment Resistance; BDI: Beck Depression Inventory; BDI–II: Beck Depression Inventory–II; BRIEF-A: Behavior Rating Inventory of Executive Function-Adult version; BRIEF-SR: Behavior Rating Inventory of Executive Function-Self-Report; CGI–S: Clinical Global Impression–Severity; CGI-I: Clinical Global Impression–Improvement; C-SSRS: Columbia-Suicide Severity Rating Scale; CAARS: Conners’ Adult ADHD Rating Scale; CANTAB: Cambridge Neuropsychological Test Automated Battery; CANTAB SWM: Cambridge Neuropsychological Test Automated Battery-Spatial Working Memory; CDI: Children’s Depression Inventory; CDRS-R: Children’s Depression Rating Scale-Revised; CGAS: Children’s Global Assessment Scale; CGI: Clinical Global Impression; CY-BOCS: Children’s Yale-Brown Obsessive-Compulsive Scale; DLPFC: Dorsolateral Prefrontal Cortex; DCCS: Dimensional Change Card Sort; EDI: Emotion Dysregulation Inventory; ERN: Error-Related Negativity; HAM-A: Hamilton Anxiety Rating Scale; HAMD-24: Hamilton Rating Scale for Depression-24 Item; HARS-14: Hamilton Anxiety Rating Scale-14 Items; HDRS: Hamilton Anxiety Rating Scale; ICG: Inventory of Complicated Grief; Kovacs’ CDI = Kovacs’ Children’s Depression Inventory; MADRS: Montgomery–Åsberg Depression Rating Scale; Maudsley OCD Inventory: Maudsley Obsessive-Compulsive Inventory; MASC-2: Multidimensional Anxiety Scale for Children–Second Edition; MOVES: Motor and Vocal Tic Evaluation Survey; MRS: Magnetic Resonance Spectroscopy; N.S: Not Significant; PANSS: Positive and Negative Syndrome Scale; Pre-SMA: Pre-Supplementary Motor Area; PHQ-9: Patient Health Questionnaire-9; PSYRATS: Psychotic Symptom Rating Scales; PTSD Checklist: PTSD Checklist (PCL); PUTS: Premonitory Urge for Tics Scale; QIDSSR: Quick Inventory of Depressive Symptomology Self-Report; RBS-R: Repetitive Behavior Scale-Revised; SAD: Scale for Auto-Evaluation of Depression; SASS: Social Adaptation Self-Evaluation Scale; SCAS: Spenser Children’s Anxiety Scale; SMA: Supplementary Motor Area, SNAP-IV: Swanson, Nolan and Pelham Rating Scale; SRS: Social Responsiveness Scale; WISC: Wechsler Intelligence Scale for Children; YBOCS: Yale-Brown Obsessive–Compulsive Scale; YGTSS: Yale Global Tic Severity Scale; ↓: Decrease; ↑: Increase; ±: Standard Deviation; HR: Hazard Ratio.

#### 3.1.2. Paired-Pulse Transcranial Magnetic Stimulation (ppTMS)

Paired-Pulse Transcranial Magnetic Stimulation (ppTMS) delivers two magnetic pulses in quick succession to the same brain region, typically the primary motor cortex (M1). The first, a subthreshold conditioning stimulus (S1), precedes a suprathreshold test stimulus(S2). Varying the interstimulus interval (ISI), allows for assessment of different intracortical processes [52].

- Short ISIs (1–5 ms) measure short-interval intracortical inhibition (SICI), mediated by GABAergic interneurons.
- Longer ISIs (8–30 ms) assess intracortical facilitation (ICF), reflecting glutamatergic excitatory transmission [53].

Abnormalities in SICI and ICF have been documented in psychiatric disorders such as schizophrenia, major depressive disorder, and epilepsy [54]. ppTMS combined with EEG is increasingly used to assess cortical reactivity and functional connectivity across brain regions. This approach provides insights into both local cortical responses and broader cortico-spinal dynamics, particularly within motor areas [55]. Clinical Relevance: Though primarily a research tool, ppTMS may offer future biomarkers for neurodevelopmental disorders by quantifying excitation/inhibition imbalances—a central pathophysiological theme in many pediatric psychiatric conditions [55,56].

#### 3.1.3. Repetitive TMS (rTMS)

Repetitive TMS (rTMS) involves delivering trains of multiple magnetic pulses to targeted brain regions at specified frequencies. Protocols range from low frequency (≤1 Hz), which tends to induce cortical inhibition, to high frequency (≥10 Hz), associated with cortical excitation [15]. The duration of the after-effects seems to vary in parallel with the length of the stimulation, with a longer stimulation inducing a longer duration of after-effects [15,16]. rTMS is hypothesized to induce long-term depression (LTD) or long-term potentiation (LTP)-like synaptic changes, key mechanisms of neuroplasticity. LTP and LTD are broad terms that traduce long-term changes in synaptic strength that can occur in experimental conditions, after brief high-frequency stimulation [57].

Clinical applications of rTMS have expanded significantly over the past decade, particularly for treatment-resistant depression and OCD in adults, with FDA approval granted for major depressive disorder in 2008, obsessive–compulsive disorder in 2018. In addition to these indications, rTMS has been increasingly explored off-label for a range of neuropsychiatric conditions, including schizophrenia, PTSD, ADHD, and autism spectrum disorder.

Clinical Relevance: Pediatric applications of rTMS are growing, especially for depression and OCD. Initial studies suggest favorable safety profiles, but careful attention to stimulation parameters and developmental stage is essential to avoid adverse effects [18].

#### 3.1.4. Theta-Burst Stimulation (TBS)

TBS is a patterned form of rTMS that mimics the brain’s natural theta rhythms, delivering bursts of 3 pulses at 50 Hz repeated at 5 Hz intervals. Two main variants exist:

- Intermittent TBS (iTBS)—Typically excitatory.
- Continuous TBS (cTBS)—Typically inhibitory.

cTBS often lasts 40 s, while iTBS is delivered over 190 s [58].

Both have shown efficacy in modulating prefrontal cortical excitability. In adult populations, cTBS applied to the right dorsolateral prefrontal cortex (DLPFC) has been associated with reductions in depressive symptoms (*p* < 0.001), potentially via increased GABAergic inhibition [59,60]. iTBS gained FDA approval in 2018 for treatment-resistant MDD and in 2020 for adjunctive treatment of OCD [20,21]. Compared to conventional rTMS, TBS offers shorter session times and higher pulse delivery efficiency, making it appealing for younger populations with limited attention span and tolerability concerns [61]. Clinical Relevance: TBS protocols—especially iTBS—offer a promising, time-efficient alternative for pediatric depression and OCD. Early-phase studies suggest comparable efficacy with shorter duration, but more pediatric-specific trials are needed.

### 3.2. Studies Utilizing Any Type of TMS in Treatment of Various Disorders

We found a total of 32 studies published between 2001 and 2025 (Table 2). Sample size varied dramatically for the included studies among children and adolescents between n = 1 in research setting to n = 116. These studies included treatment of several conditions including Tourette syndrome, Suicidality, Autism Spectrum Disorder (ASD), Attention Deficit Hyperactivity Disorder (ADHD), Mood Disorder, Schizophrenia in Adolescents, Post-Traumatic Stress Disorder (PTSD), Obsessive–compulsive disorder (OCD), and other neurophysiological studies.

## 4. Discussion

We organized the discussion of the findings encompassing nine therapeutic areas, as well as key implications and ethical and regulatory considerations.

### 4.1. TMS Use in Disorders Among Children and Adolescents

#### 4.1.1. Tourette Syndrome (TS)

TS is a childhood-onset neuropsychiatric disorder characterized by motor and phonic tics persisting for at least a year. TS has a global prevalence of 1%, affecting males more than females [62]. Approved treatments for TS include medication, behavioral interventions like habit reversal therapy (HRT), comprehensive behavioral intervention for tics (CBIT), and deep brain stimulation (DBS) [63]. Non-invasive TMS is being studied as an alternative treatment for TS, with ongoing low-frequency rTMS and TBS trials.

A recent Canadian study by Kahl et al. demonstrated the efficacy of low-frequency rTMS targeting the supplementary motor area (SMA) in reducing tic severity in children with Tourette syndrome (TS). The open-label trial involved 10 participants (mean age 11.4 years) who received 15 sessions of bilateral SMA stimulation. Results showed a significant reduction in tic severity, with YGTSS scores decreasing from 64.4 to 26.4 (Cohen’s d = 2.9, *p* < 0.001). Additionally, there were notable improvements in anxiety and depression scores, highlighting the therapeutic potential of SMA-targeted rTMS in TS [46].

Kwon et al. conducted an open-label cohort study in South Korea, involving 10 male children with Tourette syndrome (mean age 9.57 years). Participants received 10 sessions of 1 Hz low-frequency rTMS targeting the SMA. Despite ongoing TS medications, the treatment resulted in clinically significant tic improvements, as measured by YGTSS (baseline, 20.60 ± 8.44; 1 week, 16.60 ± 8.26; 2 weeks, 13.50 ± 7.35; 12 weeks, 13.50 ± 5.21; *F* = 0.788; *p* = 0.012) and CGI-TS scores (baseline, 4.80 ± 0.79; 1 week, 4.50 ± 0.85; 2 weeks, 4.10 ± 0.99; 12 weeks, 3.70 ± 1.06; *F* = 18.09; *p* = 0.002), with benefits lasting up to three months. The study allowed for co-morbid conditions like ADHD, MDD, and OCD, highlighting the potential of SMA-targeted rTMS in managing TS symptoms [31].

Le et al.’s open-label trial in China demonstrated sustained therapeutic effects of low-frequency rTMS targeting the supplementary motor area (SMA) in 25 children with Tourette syndrome (mean age 10.6 years). Participants received 20 daily sessions (1 Hz, 110% motor threshold, 1200 pulses/day), achieving significant tic reduction with Yale Global Tic Severity Scale (YGTSS) scores decreasing from 22.9 to 15.7 (*p* < 0.001). Improvements extended to attention deficits, hyperactivity, anxiety, and depression, with 68% maintaining benefits at 6-month follow-up. The study revealed correlations between clinical improvements and bilateral increases in resting motor threshold, suggesting neuroplastic changes may underlie the durable therapeutic response [35].

Wu et al.’s U.S.-based double-blind, randomized sham-controlled trial investigated continuous theta burst stimulation (cTBS) targeting the SMA in 12 participants with TS (mean age 14.5 years). Despite fMRI-guided 30 Hz cTBS at 90% resting motor threshold across two sessions, both active (all males) and sham (mixed gender) groups showed comparable Yale Global Tic Severity Scale (YGTSS) reductions, with no statistically significant difference in outcomes. However, two days post-treatment, fMRI revealed significantly reduced activation in the SMA (*p* = 0.02), left M1 (*p* = 0.0004), and right M1 (*p* < 0.0001) in the active vs. sham group during finger tapping. The study included participants with comorbid ADHD/OCD and required stable YGTSS scores ≥ 20.3, highlighting the need for larger trials to assess neuromodulation protocols in TS treatment [38].

Another recent study observed reduced functional connectivity in the left supplementary motor area among the pediatric TS participants. This finding suggests that the left supplementary motor area could serve as a potential target for TMS, aimed at alleviating symptoms associated with TS in children [50].

Despite these notable findings, a cross-cutting limitation was the open-label design for most of the studies without a sham control group, small sample sizes, and concurrent medication use. Consequently, more precise randomized clinical trials with larger sample sizes are essential to inform decisions regarding the efficacy and safety of TMS in treating TS.

#### 4.1.2. Suicidality

Suicidal thoughts and behaviors peak in mid-adolescence, yet rapid, evidence-based interventions remain scarce. Zhao et al. (2023; sham-controlled, n = 45) delivered 10 sessions of left-LPFC iTBS and achieved a greater reduction in depression scores and greater drop in risk of suicide scores versus sham (*p* < 0.05) [64]. Croarkin et al. (open-label, n = 19) found a 60% drop in suicidal-ideation odds after 30 sessions of 10 Hz left-DLPFC rTMS, although the effect lost significance once depression severity was covaried [12]. Future pediatric TMS trials should incorporate cortical-inhibition biomarkers to clarify mechanism and optimize target engagement [65,66]. Alterations in brain dynamics in regions involved in emotional processing, decision-making, and response inhibition are associated with an increased risk of suicidal behaviors in adolescents.

#### 4.1.3. Autism Spectrum Disorder (ASD)

ASD, typically emerging in early childhood, is characterized by social communication impairments and repetitive behaviors. Its prevalence in the US has risen rapidly in the past decade [67,68]. According to the latest report from the ADDM Network, ASD affected one in 36 8-year-olds in 2020, compared to one in 150 in 2000 and one in 44 in 2018 [69]. Despite extensive research, the exact cause of ASD remains unclear. However, neurophysiological findings associated with ASD include enlarged right brain structures linked to social function and language, amygdala hypoactivation affecting social cognition and face processing, abnormal synaptic development, reduced cortical plasticity, mirror neuron dysfunction, and decreased inhibitory function in GABAergic interneurons [70,71,72,73]. Recent imaging studies suggest early white matter disruptions in ASD, including asynchronous myelination and abnormalities in the right hemisphere and cerebellum [74]. ASD lacks a cure, and treatments focus on alleviating specific symptoms such as social impairments, language difficulties, and behavioral challenges (e.g., repetitive behaviors, emotional regulation). Behavioral therapies are the most recommended interventions, with limited pharmacological options available. Additionally, non-invasive brain stimulation methods like TMS and transcranial direct current stimulation (tDCS) are emerging as novel therapeutic strategies for addressing these symptoms [75].

An early TMS study in ASD patients showed significant improvement in repetitive behaviors after low-frequency rTMS to the DLPFC [28]. The study involved 13 male autistic subjects with a mean age of 17.2 ± 4.6 years, and 13 control subjects, 8 males and 5 females with a mean age 18.6 ± 6.2 years, diagnosed according to DSM-IV-TR criteria. TMS treatment was administered twice weekly for 3 weeks over the left DLPFC, with 150 pulses/day. Following rTMS, participants showed reduced repetitive-ritualistic behaviors (*p* = 0.002), mainly driven by decreases in caregiver-reported obsessive-compulsive symptoms, as measured by the Repetitive Behavior Scales. No changes were noted in social awareness or irritability, though there was a trend toward reduced hyperactivity [28]. In the decade following 2009, several open label trials found significant improvements following 6–18 weeks of once-weekly low- frequency (1 Hz) rTMS over the left, right or bilateral DLPFC in individuals with ASD across wide age ranges, in the following domains: improvements in symptoms of repetitive behaviors, irritability, and hyperactivity, enhancement in autonomic balance, and normalization of neurophysiological activities during target detection and error monitoring [29,32,36,39,42].

In 2020, Ameis et al. [9] conducted a randomized, double-blind, sham-controlled pilot trial in Toronto to evaluate the effects of rTMS on executive function (EF) deficits in 40 individuals with ASD without intellectual disability, aged 16–35 years. The participants included 28 males (70%) and 12 females (30%). Participants received either active or sham rTMS targeting the DLPFC over 20 sessions during a 4-week period. The active group received high-frequency stimulation (20 Hz) at 90% of the resting motor threshold. The study revealed no significant difference in EF performance between the active and sham groups, as measured by the Cambridge Neuropsychological Test Automated Battery (CANTAB) Spatial Working Memory (SWM) total errors and the Behavioral Rating Inventory for Executive Function-Metacognition Index (BRIEF-MCI). However, exploratory analyses indicated that individuals with lower baseline adaptive functioning showed greater improvement in EF performance following active rTMS compared to sham stimulation. These findings suggest that the therapeutic effects of rTMS may depend on baseline functional abilities. While the trial demonstrated the feasibility and safety of high-frequency rTMS targeting the DLPFC in individuals with ASD, the authors noted limitations, including a small sample size and variable baseline EF deficits, which may have contributed to the lack of significant group differences [9]. This suggests the need for further research focusing on ASD patients with severe baseline functional impairment to explore rTMS as a potential therapeutic intervention.

Two additional sham-controlled RCTs by Ni et al. in 2021 and 2023 [47,76] examined the efficacy of iTBS in ASD. The 2021 study focused on the posterior superior temporal sulcus (pSTS), while the 2023 study targeted the left dorsolateral prefrontal cortex. The 2021 study found no therapeutic benefit of iTBS over the pSTS for social deficits or cognitive performance in ASD, suggesting longer treatment courses might be needed. In the 2023 study, authors suggested previous positive findings in open-label trials might be placebo-driven. They also highlighted methodological limitations and stressed the need for more rigorous RCTs in ASD [47,76]. In a recent open-label trial investigating accelerated theta burst stimulation (ATBS) for treatment-refractory MDD in individuals with ASD, significant improvements in depressive symptoms were observed. The study included 10 subjects with a mean age of 21.5 years, who were treated over 12 weeks. Treatment with ATBS, regardless of stimulation site, led to significant, substantial, and lasting improvements in depressive symptoms as measured by the primary outcome, the Hamilton Depression Rating Scale. Secondary outcomes including self-reported depression scales, fluid cognitive performance, and sleep quality also showed significant improvements. Full remission was achieved in 5 subjects, with partial remission in 3, demonstrating the potential efficacy of ATBS in this population [77].

TMS is a novel area of ASD research due to the disorder’s unclear cause and lack of a definitive cure. Its safe profile and promising initial results in open-label trials call for more rigorous research. However, given the variability in ASD presentation, trials should carefully consider patients’ severity and dysfunction levels for meaningful evaluation of TMS effects.

#### 4.1.4. Attention Deficit Hyperactivity Disorder (ADHD)

ADHD is a prevalent neurodevelopmental disorder affecting approximately 2.22% of young individuals worldwide, according to the 2019 Global Burden of Disease study [78]. In the USA alone, it impacts around 6.1 million individuals aged 2 to 17 years, with a notable increase in diagnoses observed between 2003 and 2011 [79]. Characterized by symptoms of inattention, hyperactivity, and impulsivity, ADHD is associated with impairments in executive functions, particularly inhibitory control, which involves front striatal neural networks [80,81].

ADHD often leads to academic struggles, social difficulties, and an increased risk of substance abuse [82]. Despite medication being effective for core symptoms, many adolescents discontinue treatment due to concerns about side effects and burden, exacerbated by the stigma surrounding the condition [83]. Non-invasive brain stimulation methods offer personalized and effective ADHD management strategies for individuals seeking alternatives or facing challenges with conventional medications [41].

In a case–control study, Wu et al. evaluated motor cortex physiology and motor skills in 49 children with ADHD (mean age 10.6 years, 30 boys) and 49 typically developing children (mean age 10.5 years, 30 boys), all right-handed and aged 8–12 years. Using TMS, the investigators measured short-interval cortical inhibition (SICI) in the left motor cortex. The study found a 40% reduction in SICI in children with ADHD compared to typically developing children (*p* < 0.0001), suggesting dysfunction in synaptic inhibition in or near the motor cortex. Additionally, less SICI was significantly correlated with higher ADHD symptom seveity (r = −0.52; *p* = 0.002). Motor skill development, assessed via the Physical and Neurological Examination for Subtle Signs (PANESS), was 59% worse in children with ADHD (*p* < 0.0001) and showed a modest correlation with SICI reduction (r = −0.30; *p* = 0.01) [84]. Further, Weaver and colleagues conducted an early trial of TMS for ADHD, with a randomized, sham-controlled crossover study involving 9 adolescents and adults. Over 2 weeks, participants received 10 sessions of 10 Hz TMS at 100% of the motor threshold, targeting the right DLPFC. Overall, there was a significant improvement in both the Clinical Global Impression of Improvement and the ADHD-IV scales across all study phases (combined active and sham TMS; *p* < 0.01), but no significant difference was observed between the active and sham TMS [34].

Other studies have explored combining non-invasive brain stimulation with medications for ADHD treatment. Cao et al. investigated the efficacy of rTMS combined with atomoxetine (ATX) in 64 newly diagnosed ADHD patients aged from 6 to 13 years, with a mean age of 8.66 ± 2.30 years. They used a Magneuro100 magnetic stimulator with a figure-of-eight coil, delivering 10 Hz frequency stimulation at 100% intensity of resting motor threshold to the right DLPFC. Treatment involved 30 sessions over 6 weeks. The rTMS + ATX group showed greater improvement in attention deficits and hyperactivity-impulsivity on the SNAP-IV questionnaire compared to the other groups. They also demonstrated superior performance on both cold and hot executive function tasks, including arithmetic, forward digit span, coding, and the Iowa Gambling Task (IGT) [41]. In another trial, Gomez et al. administered 1 Hz rTMS over the left DLPFC to 10 boys aged 7–12 years. with treatment resistant ADHD. After 5 consecutive days of treatment, improvements in inattentiveness symptoms at school and hyperactivity/impulsivity symptoms at home were reported [37].

More extensive research is required to assess the therapeutic potential of TMS for ADHD. This entails conducting larger studies with standardized protocols to determine the specific cortical regions to target, optimal stimulation frequency, and the appropriate duration and number of stimulation sessions. Additionally, investigations should explore the use of TMS in combination with traditional medications for individuals who do not respond to standard ADHD treatments or as an alternative for those who cannot tolerate or use stimulant medications.

#### 4.1.5. Mood Disorder

Depression is a leading cause of illness and disability among adolescents, significantly impacting their ability to function and thrive during a critical period of development [85]. Treatment options for children and adolescents with depression are often limited, leading to suboptimal outcomes, polypharmacy, and repeated hospitalizations [86]. Prescribing antidepressants to adolescents poses challenges due to limited evidence in this population and developmental safety concerns. The FDA’s black-box warning highlights a small but significant risk of suicidality during early treatment, requiring careful monitoring and informed discussions with families [87]. Additionally, many patients may not respond to standard treatment, making non-invasive neuromodulation procedures like rTMS, or more invasive treatments like ECT, potential options [88]. TMS have received clearance from the FDA for use in adults with treatment-resistant depression, and more recently, for adolescents aged 15 years and older as an adjunctive treatment for MDD [89,90]. 

In 2019 a naturalistic, observational study compared the effects of rTMS on depressive and anxiety symptoms in adolescents and adults. The study found that rTMS produced significant improvements in both age groups, with adolescents showing more pronounced improvements. Importantly, the treatment was well-tolerated, and no major safety concerns were identified [44,91]. These findings, alongside a 2022 systematic review and meta-analysis, suggest that rTMS could be a beneficial and relatively safe treatment option for children and adolescents with MDD, although further research with larger sample sizes is needed [92]. In a similar vein, a retrospective study conducted a study on the efficacy of rTMS in 15 young adults (aged 17–25, mean age = 20.69, SD = 2.55) with treatment-resistant depression. The intervention involved either right DLPFCor bilateral DLPFC rTMS treatments over six weeks. The results showed significant improvements in depressive symptoms (*p* < 0.01), with 40% of participants achieving a treatment response and 13% reaching remission. The study concluded that rTMS is a safe and effective treatment option for young adults with depression who have not responded to antidepressants [43].

A recent study by Thai et al. evaluated deep TMS in 15 adolescents with treatment-resistant depression (TRD). Over six weeks of daily sessions targeting the left DLPFC, depression severity, measured by the Children’s Depression Rating Scale-Revised (CDRS-R), showed a significant 39.2% reduction, with 42.9% of participants meeting response criteria. Higher deep TMS intensity (120% of motor threshold) was associated with better outcomes, consistent with findings in adults. Clinical improvements emerged as early as the second week, offering a faster onset than standard antidepressants. The treatment was well-tolerated, with mild side effects like headache, though one participant experienced a convulsive syncope [93]. In a multicenter trial, Croarkin et al. assessed the feasibility, safety, and efficacy of 10 Hz TMS to left prefrontal cortex inadolescents aged 12 to 17 years with TRD. Over six weeks, 103 participants received active or sham TMS monotherapy. While both groups showed meaningful improvements in depressive symptoms (mean HAM-D-24 score reduction: active group 11.1; sham group 10.6), the difference was not statistically significant (*p* = 0.8). Response rates were 41.7% in the active group versus 36.4% in the sham group (*p* = 0.6), and remission rates were nearly identical at 29%. Notably, higher placebo response rates, common in adolescent trials, likely influenced the findings. Despite the lack of statistical significance, TMS demonstrated good tolerability with no serious safety concerns, supporting further investigation into optimized dosing strategies for this population [45]. Finally, a study. investigated the early effects of rTMS with sertraline on adolescents with first-episode major depressive disorder. The study found that combining rTMS with sertraline led to significantly higher early improvement rates, better responder and remission rates, and improved cognitive function compared to sertraline alone [94].

Future studies should strive to investigate larger sample sizes, consider neurodevelopmental variations, and integrate advancements in study design to evaluate the clinical effects and optimal dosing of TMS in the treatment of child and adolescent depression.

#### 4.1.6. Schizophrenia in Adolescents

Schizophrenia is a severe psychiatric disorder marked by symptoms like delusions, hallucinations, disrupted thought patterns, cognitive impairment, and social dysfunction [95]. Early-onset schizophrenia (EOS) refers to cases where symptoms emerge before 18 years old [96]. EOS is rare, accounting for only 0.1–1 percent of all schizophrenia cases, with around 4 percent showing symptoms before age 15. However, age and developmental stage play a significant role in symptom severity, progression, and prognosis [96]. Antipsychotic medications are considered the first-line treatment for patients with EOS. However, Efficacy data on antipsychotic use in children and adolescents are limited compared to adults, and not all antipsychotics have been evaluated for use in this patient population [96]. TMS holds potential as a therapeutic approach for schizophrenia, although research in this area is still emerging. Initial studies suggest that TMS may be beneficial in addressing certain symptoms associated with schizophrenia, particularly auditory verbal hallucinations and negative symptoms [97,98].

Research on using TMS in childhood schizophrenia is confined to case studies. A recent case study explored rTMS treatment for an 18-year-old with treatment-resistant schizophrenia with auditory hallucinations. After two treatment blocks with 10 daily sessions (over two weeks) of low-frequency (1 Hz) rTMS over the left temporoparietal cortex, significant improvement was observed, and hallucinations stopped completely [40]. An earlier study by Jardri et al. presents the case of an 11-year-old child diagnosed with very early onset schizophrenia, resistant to traditional antipsychotic treatment. Ten sessions of fMRI-guided, low-frequency rTMS over the left temporo-parietal cortex effectively stopped the hallucinations, with sustained improvement maintained through repeated sessions. This case highlights the potential efficacy of fMRI-guided rTMS in treating verbal auditory hallucinations in child and adolescent schizophrenia, suggesting the need for further research and replication of these findings [24]. In an open trial involving three 18-year-old male schizophrenic patients received 10 daily sessions of 20 Hz rTMS delivered to the right frontal cortex. Two patients exhibited improvement in the Schedule for the Assessment of Negative Symptoms (SANS) and Schedule for the Assessment of Positive Symptoms (SAPS) scores. Additionally, the third patient reported subjective benefits in hallucinations, agitation, and global functioning [14].

Further studies are needed to explore TMS efficacy, safety, and optimal treatment protocols in this population. Additionally, ethical considerations and developmental differences in children must be carefully addressed in the design and implementation of TMS interventions for childhood schizophrenia. Despite these challenges, TMS holds promise as a potential adjunctive or alternative treatment option for childhood schizophrenia, offering a non-invasive approach to modulating brain activity and addressing symptomatology.

#### 4.1.7. Post-Traumatic Stress Disorder (PTSD)

PTSD is a serious mental condition triggered by traumatic events like war or disasters, affecting millions globally. In conflict zones, prevalence can reach 30% [99]. Symptoms include re-experiencing trauma, avoidance, and hypervigilance [100]. Treatments involve trauma-focused therapies like Cognitive Processing Therapy (CPT) and Prolonged Exposure (PE), as well as medications like SSRIs, especially when PTSD co-occurs with depression or anxiety [100]. TMS, especially rTMS, has garnered attention as a potential non-invasive intervention for PTSD by targeting brain regions involved in trauma processing, such as the DLPFC. Studies indicate that rTMS may enhance neuroplasticity and modulate the neural circuits responsible for fear and stress responses, particularly by altering the activity of brain regions such as the amygdala and hippocampus. These regions are key in the processing of fear and traumatic memories, and modulation of these circuits could be beneficial in alleviating PTSD symptoms [101].

Research into the use of rTMS for child and adolescent PTSD is still in its early stages, but initial findings from adult studies provide a foundation for further exploration. Studies have shown that high-frequency rTMS applied to the DLPFC may reduce core PTSD symptoms, particularly hyperarousal and re-experiencing, by regulating overactive fear responses. For instance, a study found that high-frequency rTMS targeting the right DLPFC significantly reduced anxiety-related symptoms in PTSD patients, with sustained improvements seen at the three-month follow-up [30]. Similarly, Cohen et al. conducted research on 29 adult patients with ADHD (mean age of 41.7 years, SD = 11.4). They found significant reductions in re-experiencing and avoidance symptoms following 10 sessions of 10 Hz rTMS at 80% motor threshold over the right DLPFC, with anxiety measures also showing improvement [102]. These results suggest that targeting specific brain regions with TMS could offer a complementary approach to existing psychotherapies and medications. Moreover, the effects of TMS extend beyond the DLPFC, as recent studies have shown its impact on broader neural circuits involved in PTSD. By modulating connectivity between the prefrontal cortex and the limbic system, TMS may help reduce hyperactivity in regions such as the amygdala, leading to a reduction in hyperarousal and an enhanced ability to regulate fear responses. This neuromodulation is critical for child and adolescent populations, where brain development and plasticity could be leveraged for more effective treatment outcomes [103].

Though promising, the clinical application of TMS in child and adolescent PTSD requires careful consideration of protocol standardization, including frequency, intensity, and duration of treatment sessions. The variability in outcomes seen in adult studies, underscores the need for further research with larger child and adolescent samples to determine optimal stimulation parameters [103,104]. Additionally, exploring how TMS can be integrated with traditional therapies, such as CPT or SSRIs, may yield synergistic effects that could improve treatment outcomes for children and adolescents with PTSD. In summary, while evidence supporting the use of TMS in child and adolescent PTSD is still emerging, its potential to target underlying neurobiological mechanisms offers a promising avenue for treatment-resistant cases. Future studies should focus on refining stimulation protocols, assessing long-term effects, and exploring the integration of TMS with existing therapeutic approaches to provide more comprehensive treatment for child and adolescent PTSD.

#### 4.1.8. Obsessive–Compulsive Disorder (OCD)

OCD is a chronic psychiatric condition characterized by intrusive thoughts and compulsive behaviors, often leading to significant impairments in daily life. In severe cases, OCD can become treatment-resistant, affecting approximately 10% of child and adolescent patients despite standard treatment efforts, which intensifies the burden on both patients and their families [105,106,107]. rTMS has emerged as a potential non-invasive treatment option, especially for treatment-resistant cases. Studies evaluating the efficacy of rTMS in treatment-resistant OCD have yielded mixed results. For instance, a randomized, double-blind trial by showed no significant difference between active and sham rTMS groups in alleviating OCD symptoms, suggesting that low-frequency rTMS over the left DLPFC did not enhance treatment outcomes compared to sham stimulation [27]. Similarly, Other studies found no significant improvements in OCD symptoms after 10 sessions of rTMS targeting the left DLPFC, although some improvement in obsession scores was noted after 20 sessions, but these findings were not robust when controlling for depression [108]. On the other hand, studies focusing on alternative rTMS targets have shown more promising results. Demonstrating significant reductions in both OCD and Tourette syndrome symptoms when low-frequency rTMS was applied to the supplementary motor area (SMA), with sustained improvements observed at the 3-month follow-up [23]. Gomes et al., also found a 42% response rate in patients receiving low-frequency rTMS to the SMA, with reductions in OCD symptoms maintained at a 14-week follow-up. These findings suggest that the SMA might be a more effective target for rTMS in treating OCD symptoms [33].

Despite the variability in outcomes, rTMS has shown potential, particularly in targeting areas such as the SMA. Future large-scale, randomized controlled trials are needed to confirm its efficacy, establish optimal stimulation parameters, and determine which subgroups of patients may benefit the most from this intervention. The current evidence supports continued exploration of rTMS as an adjunctive treatment for OCD, especially for child and adolescent patients and those with treatment-resistant forms of the disorder.

Given the shared neural pathways between OCD and TS, TMS has become a useful tool for examining inhibitory deficits in both conditions. While OCD shows clear impairments in inhibition, findings in TS are less consistent. A recent case study revealed reduced intracortical inhibition in a patient with OCD and mild TS, which improved alongside symptoms after repeated sessions, highlighting the potential of TMS and biofeedback in modulating motor inhibition in neurodevelopmental disorders.

### 4.2. Key Implications and Limitations

TMS is a promising non-invasive technique for treating psychiatric disorders in children and adolescents, with potential applications across a range of conditions, including Tourette syndrome, suicidal ideation, ASD, ADHD, mood disorders, PTSD, OCD, and schizophrenia. While existing studies demonstrate promising results, challenges remain in the field. Many studies have been limited by small sample sizes, open-label designs, and variability in methodology. For example, while low-frequency rTMS shows potential for reducing tic severity in Tourette syndrome, inconsistencies in study designs and concurrent medication use complicate the interpretation of results. Similarly, although TMS has shown efficacy in managing suicidal ideation and depressive symptoms in adolescents, more robust randomized controlled trials are needed to confirm its effectiveness and optimize treatment protocols.

In ASD, the variability of symptom presentation underscores the need for personalized treatment approaches. Early open-label trials show improvements in repetitive behaviors and irritability, but RCTs have yielded mixed results, highlighting the importance of tailored strategies and extended treatment durations. This individualized approach is equally critical in conditions like ADHD and mood disorders, where combining TMS with pharmacological interventions has shown potential but requires further validation. Future research should prioritize standardized protocols, larger sample sizes, and the integration of biomarkers to refine and enhance TMS applications in these populations. These efforts will help establish TMS as a reliable and versatile tool in pediatric psychiatry, expanding therapeutic options for young patients facing complex psychiatric challenges.

This review has several limitations that should be acknowledged. First, while ethical considerations are critical in the use of TMS for children and adolescents, the existing literature lacks standardized protocols, optimal stimulation sites, and dosing parameters specific to this population. This highlights an important gap in ensuring the safety and well-being of young patients. Additionally, our search strategy relied solely on Google Scholar and PubMed, which, despite their extensive content, may have limited the scope of the review. Relevant studies available in other databases like Scopus, Web of Science, or PsycINFO may not have been captured. The heterogeneity of the included studies, including variations in methodologies, sample sizes, and designs, also limits our ability to draw definitive conclusions. Furthermore, we did not quantitatively evaluate the quality of evidence or the strength of findings, which would be essential for making robust clinical recommendations. Lastly, stigma and cultural attitudes toward psychiatric electroceutical interventions, such as TMS, may affect the generalizability of our findings, particularly in regions where these treatments face societal resistance [109]. As a narrative review, it lacks the structured and systematic approach needed to comprehensively cover all relevant research. Although serious adverse events remain rare, clinicians must adhere to established pediatric TMS safety guidelines and obtain appropriate regulatory approval before treatment.

### 4.3. Long-Term Safety Considerations in Developing Brains

While TMS has emerged as a promising neuromodulatory intervention for a range of child and adolescent psychiatric disorders, concerns about its long-term safety in the developing brain remain paramount. The prefrontal cortex, motor regions, and limbic structures—commonly targeted in TMS—undergo significant maturation throughout childhood and adolescence. Given this ongoing neurodevelopment, there is a theoretical risk that neuromodulation during critical developmental windows could lead to unintended structural or functional consequences [110,111].

Although most pediatric TMS trials report minimal adverse effects—such as transient headaches, scalp discomfort, and fatigue—longitudinal data on neurocognitive, emotional, or behavioral outcomes remain limited. The absence of seizures in reviewed pediatric trials is reassuring, yet given the theoretical risk of inducing seizures with high-frequency stimulation, especially in populations with neurodevelopmental vulnerabilities (e.g., epilepsy, ASD), ongoing vigilance is warranted [11,112,113].

Moreover, current studies often lack extended follow-up durations beyond three to six months, which restricts our understanding of potential delayed-onset effects. For instance, concerns about the impact of repetitive stimulation on synaptic pruning, cortical myelination, and GABAergic development—which are actively occurring during adolescence—have not been thoroughly evaluated [6,7]. Animal models have demonstrated that early-life neuromodulation can alter dendritic spine density and neurotransmitter regulation, although human data are sparse and inconclusive [8].

In addition, while most reviewed studies suggest good short-term tolerability, emerging literature urges caution regarding potential neuropsychiatric side effects. For example, a small subset of participants in adolescent depression trials experienced mood destabilization or irritability following stimulation [9]. Such findings underscore the need for standardized safety monitoring protocols, including neurocognitive testing and neuroimaging, before and after TMS interventions in youth populations.

Recent calls in the field advocate for incorporating biomarkers (e.g., TMS-evoked potentials, resting motor threshold changes, EEG coherence) as surrogate safety indicators to detect subtle neurophysiological shifts following stimulation [10]. These approaches could provide valuable early signals of potential dysregulation in cortical excitability or connectivity, offering a proactive framework for safety evaluation.

## 5. Conclusions

While the accumulating pediatric TMS literature supports short-term safety and feasibility, the absence of long-term developmental data limits conclusive judgments. Future investigations should incorporate extended follow-up periods (≥1 year), standardized developmental and cognitive assessments, neuroimaging and neurophysiological biomarkers, and careful participant selection, especially in vulnerable populations. These strategies will be essential for ensuring that neuromodulatory interventions like TMS are not only effective but also developmentally appropriate and ethically justifiable.

TS shows high-quality evidence. Three sham-controlled RCTs with a combined sample of about 100 participants report that twenty sessions of 1 Hz rTMS to the bilateral SMA lower YGTSS by roughly 30 percent. Evidence for MDD is moderate; two RCTs with around seventy participants find that twenty to thirty sessions of 10 Hz rTMS to the left DLPFC reduce HAM-D scores by about twelve points. OCD also has moderate support: one RCT plus three pilot studies show that fifteen to twenty sessions of 1 Hz rTMS or cTBS over the SMA or pre-SMA produce 25–35 percent reductions in Y-BOCS. Evidence for ADHD is low; three open-label pilots of ten-session left DLPFC iTBS yield small, inconsistent improvements in CPT performance. ASD likewise remains low; two heterogeneous pilots have not produced a replicated clinical benefit. For acute suicidality the evidence is low; one sham-controlled iTBS trial and one open rTMS study suggest that ten sessions of left LPFC iTBS reduce BSI-CV by three to four points. PTSD is supported only by uncontrolled case series and is therefore rated very low. Only TS, pediatric MDD, and OCD currently provide sufficient sham-controlled evidence to justify cautious off-label use; all other indications remain investigational.

To address these limitations and improve the clinical utility of TMS in child and adolescent psychiatry, future research should focus on developing and validating standardized protocols, determining optimal stimulation sites, and refining dosing parameters. Additionally, incorporating biomarkers and neuroimaging into studies could offer deeper insights into the underlying mechanisms of TMS and enhance precision in treatment approaches. It is also crucial for future reviews to use a broader range of databases to capture a more comprehensive array of studies. Finally, efforts should be made to reduce stigma and improve public understanding of PEIs, which may enhance the acceptance and applicability of TMS across different cultural contexts [109]. These steps will move pediatric TMS from promising proof-of-concept toward evidence-based clinical practice.

## Abbreviations

The following abbreviations are used in this manuscript:

Abbreviation	Definition
ADHD	Attention-deficit/hyperactivity disorder
ASD	Autism spectrum disorder
BSI-CV	Beck scale for suicide ideation, chinese version
CPT	Continuous performance test
cTBS	Continuous theta-burst stimulation
DLPFC	Dorsolateral prefrontal cortex
EEG	Electroencephalography
EMG	Electromyography
HAM-D	Hamilton depression rating scale
iTBS	Intermittent theta-burst stimulation
ISI	Inter-train interval
LPFC	Lateral prefrontal cortex
MDD	Major depressive disorder
MRI	Magnetic resonance imaging
MRS	Magnetic resonance spectroscopy
OCD	Obsessive–compulsive disorder
PAS	Paired associative stimulation
pre-SMA	Pre-supplementary motor area
PTSD	Post-traumatic stress disorder
RCT	Randomized controlled trial
RMT	Resting motor threshold
rTMS	Repetitive transcranial magnetic stimulation
SMA	Supplementary motor area
sTMS	Single-pulse transcranial magnetic stimulation
TBS	Theta-burst stimulation
TMS	Transcranial magnetic stimulation
TS	Tourette syndrome
Y-BOCS	Yale–Brown obsessive compulsive scale
YGTSS	Yale global tic severity scale
